# Pyorrhœa Alveolaris, from a Bacteriological Stand-Point, with a Report of Some Investigations, and Remarks on the Treatment

**Published:** 1899-07

**Authors:** W. J. Younger


### International Dental Journal.

Vol. XX. July, 1899. No. 7.

### Original Communications.[1]

### PYORRHŒA ALVEOLARIS, FROM A BACTERIOLOGICAL STAND-POINT, WITH A REPORT OF SOME INVESTIGATIONS, AND REMARKS ON THE TREATMENT.[2]

BY W. J. YOUNGER, M.D.

Gentlemen,—It is a customary thing among those who present papers before a learned assembly like this to preface their article by an apology for shortcomings, either from fear of the worthiness of their composition, or from an assumption of laudable modesty. I will not be so rash as to violate this hallowed precedent by omitting such an introduction to my effort, especially as I fear you will discover that my shortcomings do not belong to the fancied order, but are founded on a reality requiring your merciful consideration.

The time I had given myself for the solution of the bacteriological problem involved in the suggestion of the title of this paper has proved inadequate to the full purpose of its design. But I had a promise involved, and I felt that if I did not present to you at this meeting an introduction, at least, to the subject, which might

[1] The editor and publishers are not responsible for the views of authors of papers published in this department, nor for any claim to novelty, or otherwise, that may be made by them. No papers will be received for this department that have appeared in any other journal published in the country.

[2] Read before The New York Institute of Stomatology, April 4, 1899.

turn your attention to investigating what I believe to be the true etiology of pyorrhœa alveolaris, I should not be able by the restriction of professional labor and engagements to meet with you at all. The word "must" is a powerful one, and under its behests efforts are developed that otherwise would lie dormant, and actions achieved which ordinarily would be considered impossible. So, under the stimulus and goading of this little word, I come before you to-night, to present to you the commencement of a new line of thought which may shed a fresh light on the causation of this scourge, that has so long puzzled the wisest of our profession and been written on *ad nauseam.*

Writers have divided pyorrhœa into so many different varieties, and have called so many varied pathological conditions of the gums and alveoli by this name, that I feel it incumbent on me to define just what I personally consider pyorrhœa alveolaris to be.

Pyorrhœa alveolaris is characterized by an inflammation of the gums, a deposit of characteristic greenish-gray or slate-colored tartar, and wasting of the alveoli, accompanied by the formation of pus and pus-pockets between the tooth and alveolus; the disease being due, as I believe, to a specific bacillus. The disease is chronic in its duration, and results in the ultimate loss of the teeth. This slate-colored incrustation of which I have spoken I consider pathognomonic of this disease. There is another condition wherein we find a recession of the gums with absorption of the alveolus, and the root of the tooth is apparently free from deposit; but in these cases the gum is not swollen and inflamed, on the contrary, it is attenuated; there is no discharge of pus, and I do not think it due to a specific bacillus. The cause of this state of the gums is something entirely different. It is not true pyorrhœa alveolaris, and therefore we will not discuss the etiology of this condition in this paper.

We will now consider the formation of calculi or calcareous deposits in the body.

In order that a calculus may form, or that any deposit of earthy matter (lime salts) or crystalline substances may form, it is necessary that some insoluble substance be present to serve as a nucleus, around which layer after layer, or crystal upon crystal, can be deposited; just as a thread, placed in a thick syrup, serves to start the crystallization or deposition of sugar; so a crystal of uric acid or of triple phosphate, or a clump of mucin and bacteria, may serve as a starting-point for a deposit of the earthy matter found in solution in the tissues and fluids of the body.

The greater number of the earthy deposits found in the body, and especially those found around the teeth, are composed of calcium phosphate and calcium carbonate held together by mucin, epithelia, and bacteria. The cause of such deposits being formed around the teeth is due to the presence of bacteria, which, by a fermentative or putrefactive process, causes an agglutination of the mucin in the saliva, and this serves as a nucleus for the deposition of the earthy matter,—calcium phosphate and carbonate (tartar). In fact, none of the so-called calculi are formed anywhere in the body,—bladder, kidney, etc.,—except in the presence of bacteria (uric acid calculi being possibly excepted) which bring about a suitable condition for their formation.

In the disease which we have under consideration, we find an incrustation upon the roots of the affected teeth which differs from the ordinary calcareous tartar deposit. This dark deposit, varying in hardness, contains no bacteria, and has a nucleus or matrix composed of mucin and other proteid decomposition products of the uric acid group,—viz., xanthin, etc. In fact, from its chemistry, it could properly be classed among the xanthin calculi. The fact that no bacteria are found in the body of the deposit is of some importance in determining its formation. As already stated, the deposit is in part made up of bacterial decomposition products, which of necessity are incompatible with the life of the organism which formed them; hence, the deposit serves as a protection to the tooth substance from further invasion by bacteria, which is in keeping with the conditions found in pyorrhœa alveolaris. This fact also indicates the agency of a specific bacterium, capable of forming a specific product from the proteid substances acted upon, which is impervious to the action of the acids and other substances formed by the bacterium, which generally acts upon bony tissue. Hence, we find that the roots of the teeth are not affected chemically by these acid products. Further, the deposit, when once attached to the cementum of the root, serves as a nucleus for the deposition of the lime salts and other inorganic substance set free by the bacteria in their action on the structures around the affected tooth. This action extends to the alveolar processes, which are slowly eaten away by the acid formation of the bacteria; hence the term, though incorrect, "absorption of the alveolus." This necessarily causes a separation of the alveolus from the root, which results in loosening and eventually the exfoliation of the affected tooth.

In proof of the claim which I make that this disease is due to a specific bacterium, I beg leave to submit for your consideration the results of a bacteriological investigation made in conjunction with Dr. G. W. Cook, of Chicago, upon eight patients suffering with well-marked cases of this disease.

Before making any examination for bacteria in these cases the teeth and gums were thoroughly washed, first with methyl alcohol, forty-per-cent. strength, and then with a carbolic acid solution, five-per-cent. strength. The teeth were, moreover, wiped with cotton saturated with these solutions, reaching well underneath the gingival margin, after which mouth and teeth were washed with sterilized water. Two operations were made in each case: First, a search for the presumed specific micro-organism in the deep pus and fresh deposit; second, the removal of the calcareous deposit for separate examination. In the search for the specific bacterium a delicate, needle-shaped instrument, bent at the point, was used. It was bent in order to determine the point where the deposit ceased, in order not to pass abruptly its margin and wound the inflamed tissue beyond, and thus liberate blood whose leucocytes might destroy the germs we were in search of, and also to enable us to scrape the upper margin of the deposit, as it would there be the freshest. By this means we were able to reach the place where the germ should be in the greatest quantity and greatest activity. The instrument was first dipped in alcohol and passed through alcohol flame, and then the point was carried lightly along the calcareous surface until the end of the deposit was reached, where, with the utmost caution, it was manipulated so as to secure whatever was there. The instrument was then as carefully withdrawn as it had been inserted, the point being so held that it would not scrape the incrustation. It was from the catch at these several points that the particular cultures were made, and, as can be seen, no contamination, either from blood or the secretions of the mouth, could have been effected. A microscopical examination of the calcareous deposit revealed no bacteria in the centre, but along the outer layer various kinds of micro-organisms, both those normal to the mouth and the common pus-forming variety, were found. In the pus (taken from the deep parts, the location where active destruction of the alveoli was going on) numerous bacteria were found. Cultures were made from the tartar deposit as well as from the pus. Nothing of importance was found in the tartar deposits, but from the pus, after plating carefully in gelatin, in Petri dishes, in seven cases out of the eight a distinct bacillus, as yet undescribed, was found. To determine definitely the identity of this bacillus it was carried through the regular forms of bacteriological examination, raised in colonies, inoculated into gelatin, agar-agar, and blood-serum. In the gelatin plates colonies were developed in three or four days, which were characterized by an irregular margin, somewhat oval in shape, and raised, similar to a vesicle, upon the surface. In a few days this vesicle became a membrane and the gelatin was liquefied.

The general characteristics of this bacillus are these: In gelatin it is a very short, round bacillus, slightly motile at body temperature, 98.5° F. At ordinary room temperature, 70°, it has but little movement. It is a facultative aërobic, growing either when exposed to the oxygen of the atmosphere or deprived of it. It liquefies gelatin. In agar-agar it forms a brownish coating, but grows readily and freely in this medium. In blood-serum the bacillus seems to take on a stronger growth, appears in chain-like forms, as I show you to-night under the microscope, and after ten or twelve days apparently produces spores. As I have said, in gelatin it is a very short bacillus, and is with difficulty distinguished, in its early growth, from the coccus form. It also forms lactic acid. Three experiments have been made on rabbits with the different cultures of this bacillus,—blood-serum, gelatin, and agar-agar. All were made sick, but the one injected with the blood-serum became much more affected than the other two. In twelve days an abscess opened, and, on examination, the pus revealed the same bacillus that had been injected into it. On the sixteenth day the rabbit died, but as this occurred the evening before I left, there was no time to obtain the result of a post-mortem. The other two rabbits are still sickly, stiff in the joints, and indisposed to move, but no abscesses have as yet developed. The inoculations were all made on the same day. This seems to prove that the culture in the blood-serum was much more virulent than those in the other nutrients. It is to be regretted that so far we have been unable to inoculate human beings, but enough has been learned to satisfy us in this at least: That we have a distinct, specific bacillus found in these true cases of pyorrhœa, differentiated as a pure culture; that it is not what is known in bacteriology as a distinct pus-former, and that it is found only in those diseased portions which are not contaminated by septic material; in other words, it is, in my opinion, one of the normal bacteria of the mouth, rendered virulent through some agency which is not yet determined, which, through some trifling traumatism, gains access to the root of a tooth, and slowly and surely progresses in its destructive action until the utility of the tooth is lost. This bacillus is not found along the alveolar edge of the tooth; it is not found in the collections of tartar along the gingival margins; it is found only in the deep-seated pockets where the advance guard of the disease is progressing.

I, therefore, make these claims in regard to pyorrhœa: First, that it is a disease bacterial in origin; and, secondly, that it is a local disease, and that its pathological changes are due to a specific bacillus, and are not due to a constitutional affection. The pus found in pyorrhœa is peculiar and characteristic. It does not contain the virulent streptococci and staphylococci or leptothrix so commonly found in the ordinary ulcerations of the mouth. Where the streptococci are found it is of the brevis or non-pathogenic variety, and the pus contains lime salts, epithelial cells, serum, and the xanthin bases produced by the action of bacteria on proteid substances.

We do not for one moment deny that anything that may lower the vitality of the patient will have its effect upon the alveoli affected with this disease, as it will have its effect on all of the organs of the body. In support of this very claim I would take the liberty of citing a case, showing, as I believe, that anything which impairs the general nutritive functions or vitality of the system increases the virulency of this disease.

In June, 1896, a lady, nearly sixty years of age, came to me with one of the worst cases of pyorrhœa I ever had to contend with. Her incisors were especially bad, the upper ones projecting more than half their length from their sockets, retained only by apical attachments, and so protruding that she could not draw her upper lip over their cutting edges. In time I cured these teeth and had them back in their original position, so that but a line of the cervical margin of the roots was in evidence of the disease that had affected them. Treatment also induced a perfect attachment of the alveolar tissues with the roots. The retaining splint was removed a year ago last June, and they remain rigid in their sockets to-day. So much for the upper teeth. Before I succeeded in making the lower teeth firm, her husband, to whom she was very much attached, was taken seriously ill with heart trouble, and, after lingering a few months, died. The anxiety, loss of rest, and constant care she gave him, followed by trouble in the settlement of the estate, produced a marked general depression of the system, and reduced her weight thirty pounds.

The lower teeth had not up to this time become firm. The new tissue, not having become properly organized under this general depression, was broken down and absorbed, and there was a further recession of the gums. I am now waiting, hoping that the strength which is slowly coming back will revive the energy in the tissues of the alveolar structure, and that the same happy result which was obtained in the upper teeth may appear in the lower.

The disease has the appearance of being infectious, in that it is such a general scourge, and I think that fully ninety per cent. of the adult Anglo-Saxon race have pyorrhœa in some stage of development in one or more of the alveoli. It is common among all races, apparently, in all countries, and among all classes; the rich and the poor, the well-conditioned and the mean, the vegetarian and the meat-eater, the bibulous and the abstemious, the fat and the lean, the robust and the delicate, the strong and the weak, all are affected. Neither does temperament seem to produce immunity, for the nervous, the sanguine, and the phlegmatic suffer it. The strong argument against the theory of infection is that it often happens that only a few teeth, sometimes only one in a whole set, are affected, and continues so to the eliminating period, while the other teeth remain unaffected. If pyorrhœa were infectious, it seems to me that the inception of the disease in one tooth would be followed by the communication of the disease to all the teeth.

We all know that various theories have been offered as to the cause of this disease, and I deem it but right and fair that I should briefly call to your notice the principal ones.

Reeves was the first to bring out the uric acid theory. He claimed that nearly all of the deposits around the teeth were of uric acid origin.

Newland Pedley claimed that pyorrhœa is essentially of constitutional origin, because, as he said, both in man and the lower animals it is found connected with wasting diseases and a depressed condition of the system. Bland Sutton also seemed to claim this, having observed it in cases of rheumatism. Miller is likewise of the opinion that it is constitutional.

Patterson claimed that catarrh of the mucous membrane of the nose and pharynx and pyorrhœa alveolaris are identical. F. J. Bennett holds the same opinion.

Dr. Taft, of Ann Arbor, claims that pyorrhœa arises in consequence of a general disorder of the health. Dr. Peirce claims that it is of uric acid origin. Dr. Rhein, in a paper before the Chicago

Dental Society, at a recent meeting, claimed that it was constitutional.

In a paper read before the Oral Section of the International Medical Congress at Moscow, a well-known writer and colleague of mine, Dr. Talbot, of Chicago, cited in proof of his theory that pyorrhœa was constitutional, the effects both of syphilis and scurvy. Taking the stand that I do, that the disease is bacterial in origin and local in character, I cannot understand by what process of reasoning he connects these diseases with pyorrhœa, unless it appears to him that every pathological condition or traumatic effect on the alveoli which loosens the teeth is pyorrhœa. I mention this in particular, because not a few of our profession have labored under the same general impression, and this argument, syphilis and scurvy, has been commonly used.

Syphilis does not cause a deposit of lime salts on the root of a tooth. That the tooth loosens it is true, but not from the slow destruction of the alveolar process with its soft contents and peridental membrane, beginning at the cervical margin and eating its way slowly along the shaft of the root towards the apex, but because of the cutting off of all circulation and the consequent death of the surrounding tissues and of the pulp itself, due to syphilitic endarteritis. In pyorrhœa the bony alveolus crumbles away as the disease pervades it; in syphilis the skeleton of the alveolus remains intact and is exfoliated *en masse* or in fragments. In scurvy the same lack of deposits of true alveolar tartar on the roots is shown as in syphilis, and there is a marked difference in the general pathological conditions. Whenever a deposit of true pyorrhœal tartar is found on the root of a syphilitic or scorbutic case, you may rest assured that the incrustation was there before the advent of either syphilis or scurvy.

We will now take up the men who have looked at this disease from a *local* stand-point.

Witzel claims that it is a necrosis of the margin of the alveolus, caused by septic irritation.

Arkövy is of the opinion that the margin of the alveolus is the seat of the primary lesion. He regards it as a suppurative inflammation, beginning at the alveolar margin and spreading to the adjacent tissues.

Magitot believed it to be of parasitic nature.

Malassey and Galippe have made very careful investigations along the lines of the etiology of pyorrhœa. Galippe claims that this disease without a doubt is of local infectious origin. He bases his opinion upon investigations along local pathological lines.

D. Whittle claims the invariable presence of a micro-organism in pyorrhœa alveolaris, but without any discussion in detail, except that he considers it anaërobic.

Pyorrhœa is of slow formation and progress, requiring from five to twenty-five years or more to complete the entire waste, which results in the elimination of the tooth. This may be due to the lack of energy in the bacteria, or to a peculiar resisting power in the alveolar structure. It is as yet an undetermined point, but from my studies and observations I think the latter is the truth.

It may seem, gentlemen, that I am very dogmatic in what I state as to what I believe to be the causation of this disease, but my opinions are founded on the care and treatment of a great number of cases and a study of this disease extending over very many years. So far treatises on pyorrhœa have been to a great extent speculative individual opinions, founded on more or less observation, ofttimes considerably aided by imagination. I believe it is high time that, by the aid of bacteriological research, investigation, and experimentation, we should arrive at positive conclusions, and establish, if we are able, the true nature of this unique disease from which so many are suffering. The field is a large one, too large for a single paper, and we are at present only on the threshold. Yet, in my opinion, any effort, conscientiously made by competent observers, can but aid in the solution of this difficult problem.

*Treatment.*—The treatment of pyorrhœa involves, first, the removal of every particle of the deposit; secondly, the maintenance of the tooth in rigid position while the process of repair around the root is going on; in other words, until the complete proliferation, attachment, and thorough organization of tissue has been established; and, thirdly, the restoration of attachment between the soft tissues of the alveolus and the root.

The first can only be effected by mechanical means,—that is, by the use of instruments,—because there is no chemical as yet known, or at least that I am aware of, which would dissolve the calcareous deposit that would not also be destructive of the tissues of the alveolus. It is in the thorough removal of these deposits that the secret of success lies in the treatment of this disease, for if but one small speck is left, even though it could be framed in the point of a pin, the irritation and bacterial infection maintained by its presence would, I think, prevent the diseased surfaces from healing. It is in the detection and removal of these minute points that the skill and delicacy of touch are so much required. Great care should be taken not to wound the tissue, for by doing so you are likely to propagate trouble. The instruments, moreover, should be kept, while in use, in a sterilizing solution, not only to avoid carrying infection in case of laceration, but to protect yourself in case of puncture. I have myself been a victim twice for partially neglecting some of these precautions. Immediately that any fragment has been detached it should be flushed out by some warm antiseptic solution. In fact, before attempting to introduce a scaler, every pocket of pus should be well syringed out with some antiseptic. After the calculus has been removed the pus-pocket should be flooded with warm lactic acid, full strength, care being taken that it does not run over the margin of the pocket, on account of its very irritating nature. This can be accomplished by the careful use of absorbent cotton. The purpose of using this acid is to induce union between the soft alveolar structures and the substance of the root of the tooth. It seems to effect this union by destroying whatever necrotic tissue there may be in the sac or pocket, removing the surface of the pocket and the minute plugs which close up the mouths of the canaliculi, thereby exposing the delicate endosteal lining of the walls of these microscopic canals, and stimulating all surfaces to the proliferation of their characteristic tissue. It is my belief that the nature of this attachment between the soft tissues of the alveolar wall and the root of the tooth is similar to, while it may not be identical with, the tissues which existed before the invasion of the disease that caused their separation.

Lactic acid does not seem to act chemically on the calculus. I was led to use this acid in the expectation that it would dissolve whatever minute particles might escape the scaler, knowing that it had a strong affinity for lime salts; but after I had used it for some time I determined to make a test-tube experiment, and found that at the normal temperature of the human body a piece of tartar immersed in a vial of pure lactic acid, at the end of three months, had not diminished appreciably in size. I, therefore, discarded it for a time as a useless and deceiving agent. Later on I observed, clinically, that in all the cases in which I had used this acid the gum tissue was firmly attached to the root of the tooth, an effect which I had not been able to obtain by the use of any other agent, with the exception possibly of liquor ammoniæ fortior. I then realized the true nature of the action of this acid in these cases, and since that time it has been the one agent that I have used in the treatment of the disease, after the removal of the calculus. In further proof of the wonderful effect of this acid in stimulating granulations or proliferation of the gum tissue, I will take the liberty of citing a case: It is that of Mr. C. I had cured this gentleman of pyorrhœa, but his lower teeth were still in the embrace of ligatures waiting for them to become rigid, when he suffered from an attack of asthma complicated with other organic disturbances. One day last January he rushed into my office exclaiming, "Look, doctor, what has happened?" To my astonishment and dismay, I found, on opening his mouth, that the labial and lingual walls of the gum were entirely detached, and the interdental tissues or septa, both hard and soft, and the alveolar bone, two-thirds of the way down, were entirely gone, so that when the lip was pulled forward and the tongue retracted the teeth stood, or rather leaned, in all directions, simply being held by their apical attachment. I then ligated the teeth and held them in proper position, cleaned the roots thoroughly, rubbed them and the gums with lactic acid, and cautioned him to keep the tongue and lower lip in perfect repose for at least forty-eight hours. At the end of that time I found, on examination, that the granulations had filled up the interspaces almost to the cervices of the teeth, and the two walls of gum were bound together by them. When I examined him, two weeks ago, I found the granulations perfectly organized, and no one would have suspected the severe lesion that had occurred.

And now, gentlemen, thanking you for your kind attention to my paper, I beg leave to present to you for examination a specimen of the bacillus found, shown in blood-serum, under the microscope; also some of the pus taken from the abscess in the rabbit.

For the information in regard to the opinions of the gentlemen quoted in this paper, I am chiefly indebted to the work of our distinguished countryman and colleague, Professor W. D. Miller, of Berlin, "Micro-Organisms of the Human Mouth."

